# Supplementary material for: Serum Kynurenic Acid and Kynurenine Are Negatively Associated with the Risk of Adult Moyamoya Disease
Source: J Clin Med. 2022 Nov 29;11(23):7069. doi: 10.3390/jcm11237069 (PMC9739245; doi:10.3390/jcm11237069)
Supplement: Supplementary file 1 [file jcm-11-07069-s001.zip › jcm-2025866-supplementary.pdf]

Supplementary materials

Serum Kynurenic Acid and Kynurenine Are Negatively Associated with the Risk of Adult Moyamoya Disease

Figure S1. Flow diagram of the study participants.

Figure S2. Stratified analyses of the association between KYN (≥502.53 ng/mL versus <502.53 ng/mL) and the risk of overall MMD.

Figure S3. Stratified analyses of the association between KYN (≥502.53 ng/mL versus <502.53 ng/mL) and the risk of ischemic MMD.

Figure S4. Stratified analyses of the association between KYN (≥502.53 ng/mL versus <502.53 ng/mL) and the risk of hemorrhagic MMD.

Figure S5. Stratified analyses of the association between KYNA (≥7.65 ng/mL versus <7.65 ng/mL) and the risk of hemorrhagic MMD in the overall MMD cases.

Table S1. The association between KYNA and the risk of hemorrhagic MMD in the overall MMD cases.

Table S2. Reclassification and discrimination statistics for risk of MMD by serum KYNA or KYN.

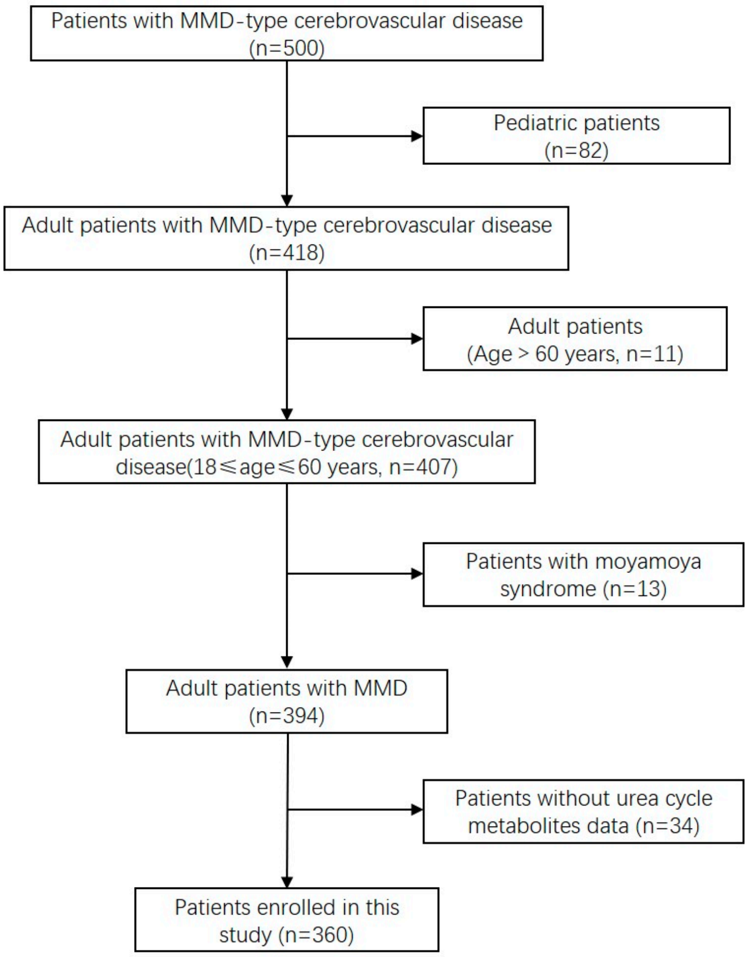

Figure S1. Flow diagram of the study participants. MMD indicates moyamoya disease; KYN, kynurenine; KYNA, kynurenic acid.

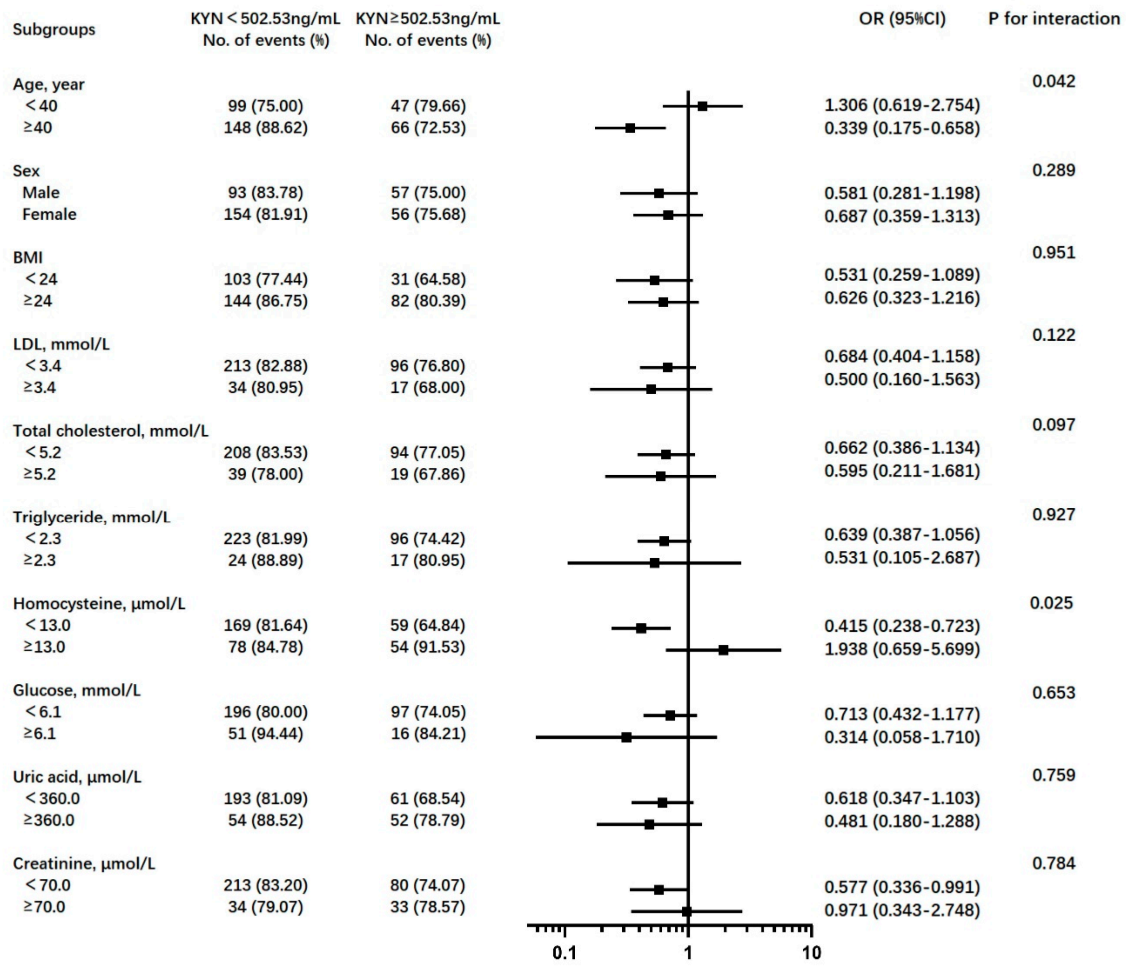

Figure S2. Stratified analyses of the association between KYN (≥502.53 ng/mL versus <502.53 ng/mL) and the risk of overall MMD. KYN indicates kynurenine; BMI, body mass index; LDL, low-density lipoprotein; OR, odd ratio.

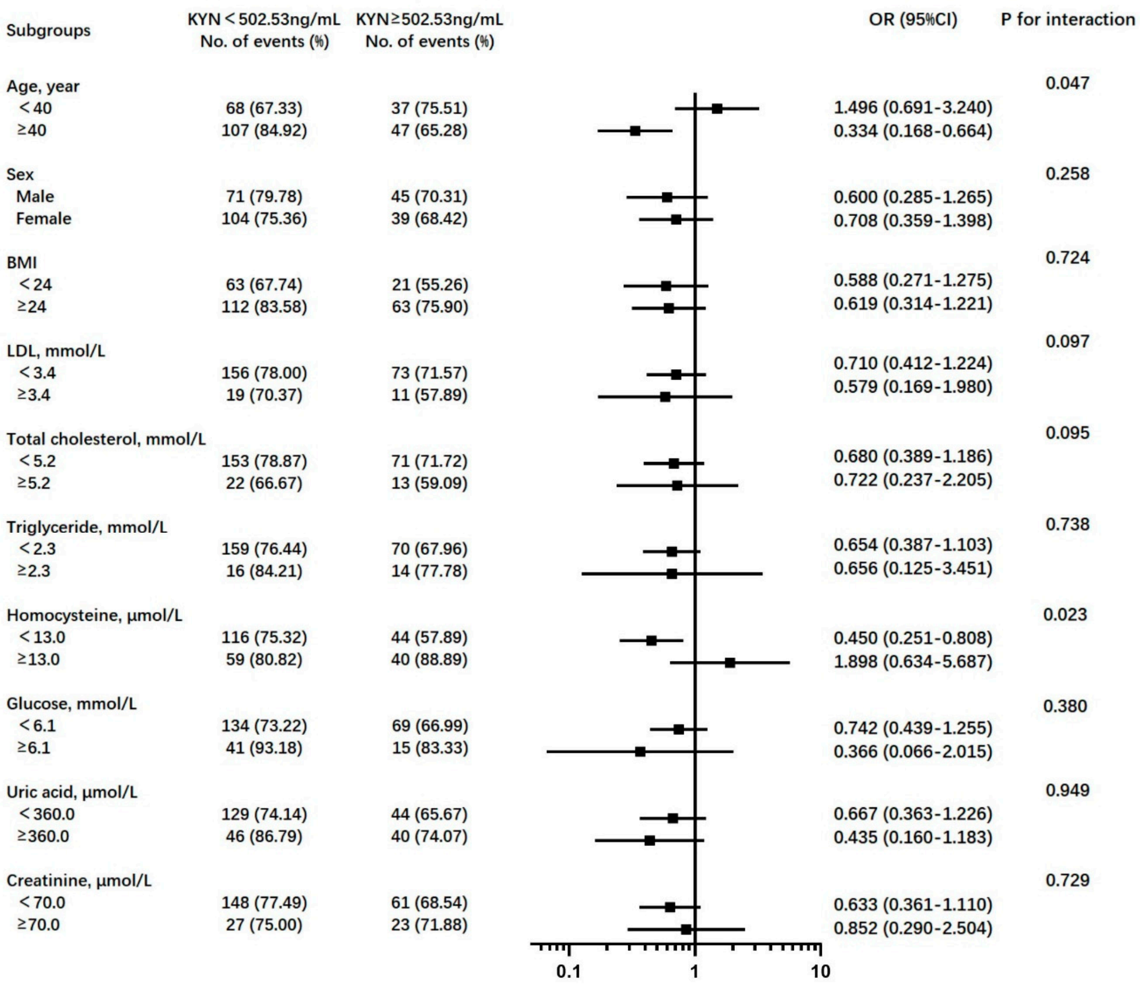

Figure S3. Stratified analyses of the association between KYN (≥502.53 ng/mL versus <502.53 ng/mL) and the risk of ischemic MMD. KYN indicates kynurenine; BMI, body mass index; LDL, low-density lipoprotein; OR, odd ratio.

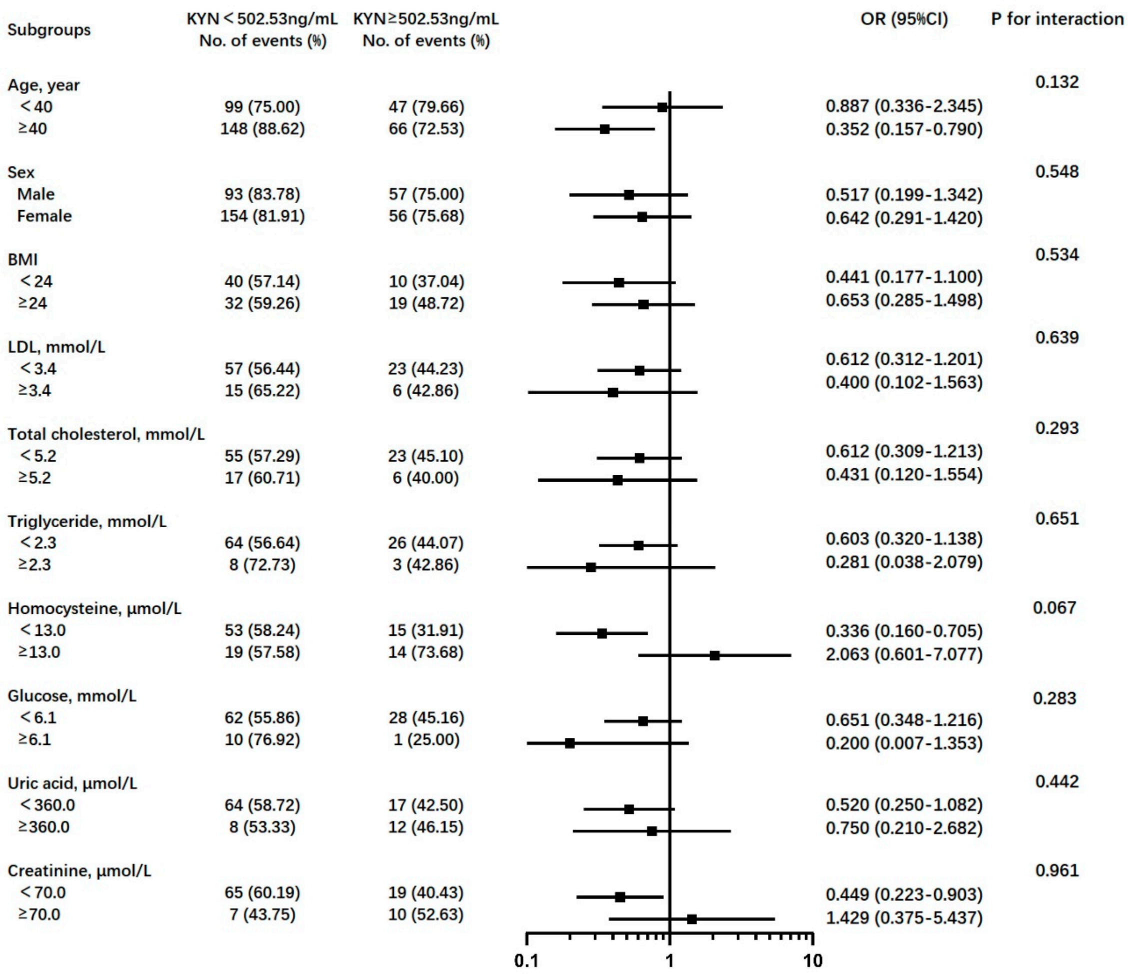

Figure S4. Stratified analyses of the association between KYN (≥502.53 ng/mL versus <502.53 ng/mL) and the risk of hemorrhagic MMD. KYN indicates kynurenine; BMI, body mass index; LDL, low-density lipoprotein; OR, odd ratio.

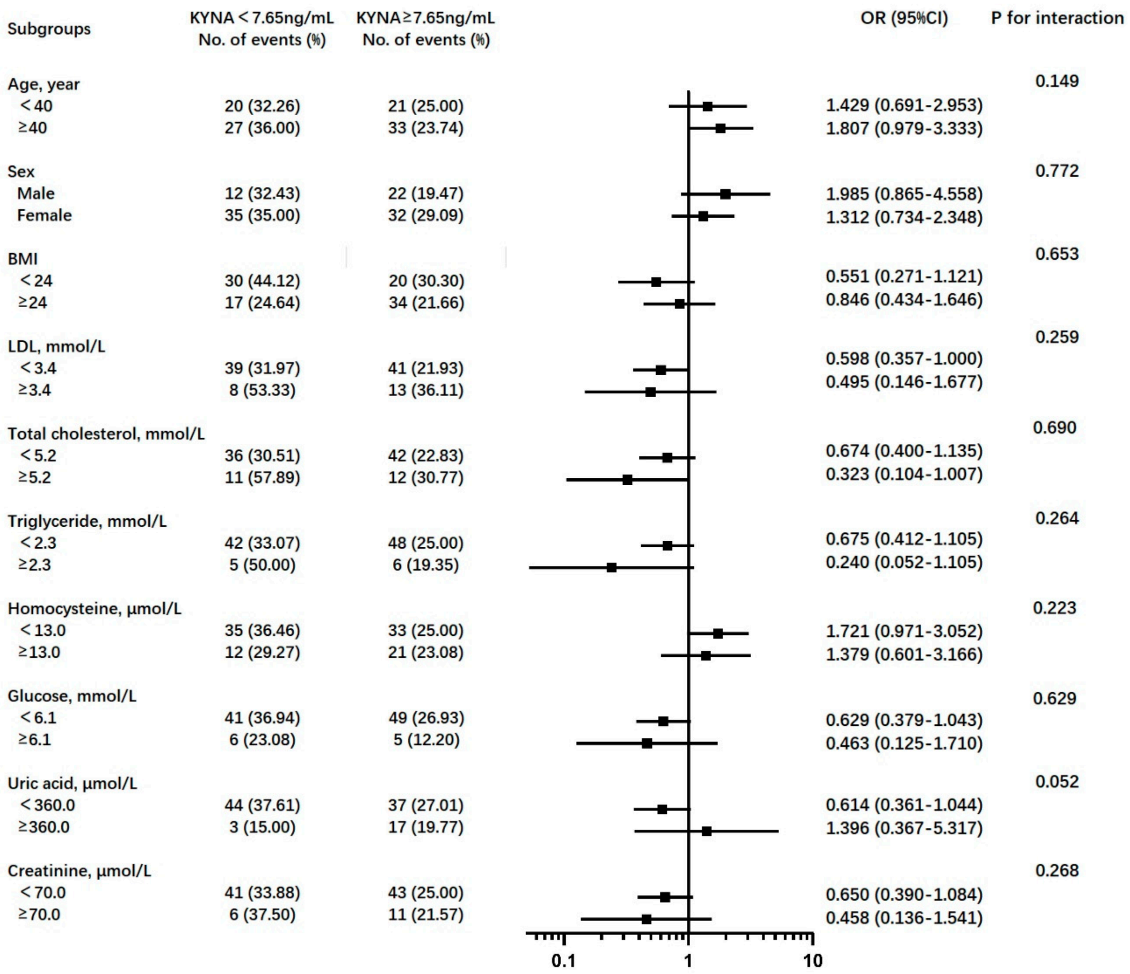

Figure S5. Stratified analyses of the association between KYNA (≥7.65 ng/mL versus <7.65 ng/mL) and the risk of hemorrhagic MMD in the overall MMD cases. KYNA indicates kynurenic acid; BMI, body mass index; LDL, low-density lipoprotein; OR, odd ratio.

Table S1. The association between KYNA and the risk of hemorrhagic MMD in the overall MMD cases.

kynurenic acid, ng/mL	No. of events (%)	Crude		Model 3*		Model 4†	
		OR (95% CI)	*P* Value	OR (95% CI)	*P* Value	OR (95% CI)	*P* Value
**Hemorrhagic MMD**	101 (28.06)						
Categories							
Tertiles							
T1 (<7.65)	47 (79.66)	1.0 (Ref)		1.0 (Ref)		1.0 (Ref)	
T2 (7.65-<11.55)	27 (47.37)	0.556 (0.319-0.968)	**0.038**	0.556 (0.313-0.988)	**0.045**	0.518 (0.280-0.958)	**0.036**
T3 (≥11.55)	27 (36.49)	0.680 (0.387-1.195)	0.180	0.743 (0.412-1.338)	0.322	0.666 (0.358-1.239)	0.199
P for trend		0.131		0.251		0.167	
T1 (<7.65)	47 (79.66)	1.0 (Ref)		1.0 (Ref)		1.0 (Ref)	
T2-3 (≥7.65)	54 (41.22)	0.612 (0.383-0.976)	**0.039**	0.636 (0.390-1.037)	0.070	0.584 (0.345-0.987)	**0.044**

MMD indicates moyamoya disease; OR, odds ratio. *Model 3 was adjusted for age, gender, WBC count, and platelet count. †Model 4 was adjusted for all the variables in model 1 plus total cholesterol, triglyceride, and LDL-C.

Table S2. Reclassification and discrimination statistics for risk of MMD by serum KYNA or KYN.

	**Continuous NRI, %**		**IDI, %**	
	**Estimate (95% CI)**	***P* Value**	**Estimate (95% CI)**	***P* Value**
MMD overall				
Conventional model	1.0 (Ref)		1.0 (Ref)	
Conventional model + kynurenic acid tertile	73.24 (54.25-92.23)	**0.000**	9.60 (6.28-12.91)	**0.000**
Conventional model	1.0 (Ref)		1.0 (Ref)	
Conventional model + kynurenine tertile	21.36 (-1.52-44.25)	0.067	1.70 (0.10-3.29)	**0.037**
Ischemic MMD				
Conventional model	1.0 (Ref)		1.0 (Ref)	
Conventional model + kynurenic acid tertile	82.05 (62.70-101.40)	**0.000**	8.72 (5.23-12.21)	**0.000**
Conventional model	1.0 (Ref)		1.0 (Ref)	
Conventional model + kynurenine tertile	23.48 (-0.16-47.12)	0.052	1.76 (0.10-3.47)	**0.043**
Hemorrhagic MMD				
Conventional model	1.0 (Ref)		1.0 (Ref)	
Conventional model + kynurenic acid tertile	116.02 (94.09-137.95)	**0.000**	31.42 (25.03-37.81)	**0.000**
Conventional model	1.0 (Ref)		1.0 (Ref)	
Conventional model + kynurenine tertile	13.39 (-14.47-41.26)	0.346	1.6 (-0.24-3.45)	0.089

MMD indicates moyamoya disease; NRI, net reclassification improvement; IDI, integrated discrimination improvement; OR, odds ratio. Conventional model included age, gender, heart rate, SBP, DBP, BMI, WBC count, lymphocyte count, platelet count, glucose, creatinine, uric acid, albumin, triglyceride, total cholesterol, HDL-C, LDL-C, apoA, apoB, and homocysteine.
